# Prevalence of acute diarrhea and associated factors among children under five in semi-urban areas of northeastern Ethiopia

**DOI:** 10.1186/s12887-021-02762-5

**Published:** 2021-06-26

**Authors:** Tarikuwa Natnael, Mistir Lingerew, Metadel Adane

**Affiliations:** Department of Environmental Health, College of Medicine and Health Sciences, Wollo University, Dessie, Ethiopia

**Keywords:** Acute diarrhea, Prevalence, Associated factors, Under-five children, Semi-urban areas, Ethiopia

## Abstract

**Background:**

Diarrheal disease is still one of the most common causes of mortality and morbidity in children under five in developing countries, including Ethiopia. Lack of specific data on the prevalence of acute diarrhea and associated factors among under-five children in the semi-urban areas of Gelsha, found in northeastern Ethiopia’s South Wollo zone, remains a major gap. Therefore, this study was designed to provide data that is important for proper planning of intervention measures to reduce the problem in this area.

**Methods:**

A community-based cross-sectional study was conducted among 340 systematically selected children under five in semi-urban areas of Gelsha from January to March 2019. The data was collected using a structured questionnaire and an observational checklist. Bivariable (crude odds ratio [COR]) and multivariable analysis (adjusted odds ratio [AOR]) were employed using binary logistic regression model with 95% CI (confidence interval). Variables with a *p-*value < 0.05 from the multivariable analysis were declared as factors significantly associated with acute diarrhea.

**Result:**

The prevalence of acute diarrhea among children under five in the study area was 11% (95%CI: 7.8–14.3%). About two-thirds (63.60%) of study participants used water from improved sources. About half (54.90%) of study participants practiced poor handwashing and 45.10% practiced good handwashing. We found that factors significantly associated with acute diarrhea were a child’s age of 12–23 months (AOR = 4.68, 95% CI: 1.45–1.50), the presence of two or more under-five children in the house (AOR = 2.84, 95% CI: 1.19–6.81), unimproved water sources (AOR = 2.97, 95% CI: 1.28–6.87) and presence of feces around the pit hole/slab/floor of the latrine (AOR = 3.34, 95% CI: 1.34–8.31).

**Conclusion:**

The prevalence of acute diarrhea among children under five was relatively high. To reduce the problem, various prevention strategies are essential, such as the provision of health education to mothers/caregivers that focuses on keeping sanitation facilities clean and child care, and construction of improved water sources. Furthermore, implementing a strong health extension program, advocating an open defecation-free environment, and practicing a community-led total sanitation and hygiene approach might be helpful to sustainably reduce childhood diarrhea.

**Supplementary Information:**

The online version contains supplementary material available at 10.1186/s12887-021-02762-5.

## Background

Acute diarrhea can be caused by different types of pathogens, among them the major five enteric pathogens *Rotavirus* (67.6%), *Adenovirus* (41.5%), enterotoxigenic *E. coli* (40.7%), *Salmonella* (38.4%), and *Giardia* (37.0%) [1]. Globally, acute diarrhea is the leading cause of morbidity and mortality among under-five children. In 2015, acute diarrhea and pneumonia accounted for almost one in every four deaths among under-five children worldwide [2]. During 2013, out of 6.3 million children who died before they reached their fifth birthday worldwide, about half (3.2 million) died from infectious diseases, with acute diarrhea killing more than 500,000 of them. It is estimated that in 2030, infectious diseases will cause 4.4 million deaths annually among under-five children, and 60% of those deaths will occur in sub-Saharan Africa [3].

A study that was conducted in the Eastern African countries of Burundi, Rwanda, and Tanzania among under-five children revealed that the prevalence of acute diarrhea was 24.80, 13.1 and 13.91%, respectively, and was associated with water- and sanitation-related factors [4]. A recent meta-analysis in three East African countries, Ethiopia, Kenya, and Somalia, from 2012 to 2017 also revealed that the average prevalence of acute diarrhea among under-five children was 27% [5]. In another study conducted in Senegal, the prevalence of acute diarrhea among under-five children was 26%, with the highest acute diarrhea prevalence in the peri-central 44.8% and urban central zones 36.3% [6].

In Ethiopia, acute diarrhea is one of the major contributors to the morbidity of under-five children [7]. The recent Ethiopian DHS 2016 report also showed a 12% prevalence of acute diarrhea at the national level [8]. According to this report, the prevalence of acute diarrhea increases from 8% among children younger than 6 months, an age when many infants are exclusively breastfeed, to 23% among those between 6 and 11 months, the age children start consuming complementary foods and other liquids. Prevalence remains high (18%) at age 12–23 months since that is the time when children start walking and coming in contact with contaminants from the environment.

According to a study conducted in Wolita Soddo town in Southern Ethiopia, the prevalence of acute diarrhea was 11% [9]. In another study conducted in rural *kebeles* of Adama District, the prevalence of acute diarrhea was 14.7% [10]. In a study conducted in a rural area of North Gondar, the prevalence of acute diarrhea was 22.1% among under-five children [11]. Despite several studies being conducted on the prevalence of acute diarrhea among under-five children in different areas of Ethiopia [9–12], there was a scarcity of evidence from semi-urban areas; one study in Enderta district in northern Ethiopia found the prevalence of acute diarrhea to be high (35.6%) in a semi-urban area [13].

The information obtained from Gelsha Health Center indicates that acute diarrhea is the major public health problem locally and is among the top 10 causes of morbidity among under-five children in that area. However, there is a lack of research on the magnitude of and factors associated with the problem among this age group in Gelsha. Therefore, this study aims to address these gaps by determining the prevalence and associated factors of acute diarrhea among under-five children in semi-urban areas of Gelsha**,** northeastern Ethiopia.

## Methods and materials

### Study area description

This study was conducted in semi-urban *kebeles* (Ethiopia’s smallest administrative unit, each consisting of about 5000 people) of Gelsha, a town found in Dessie Zuria *district*. Semi-urban areas were defined as containing  a population of 2000 or more, where roughly 20% of the total population engage in an occupation other than agriculture [13].

Gelsha is located 25 km from the city of Dessie. Dessie is the main town of South Wollo Zone, in the Amhara Region, 520 km north of Addis Ababa, Ethiopia. Gelsha has a total population of 8192, of which 4175 are men and 4017 women. Topographically, the town is mountainous (47%), plain (40%) and valley (13%). Administratively, the town is subdivided into 4 *kebeles*, of which one is semi-urban and the others rural. Agriculture is the major economic activity in the area. The town has one health center and one health post. Based on the data organized by health extension workers, the total number of under-five children in the town at the time of data collection was 770.

### Study design and source populations

A community-based cross-sectional study was conducted from January to March 2019. The source population was all under-five children paired with their mothers/caregivers in semi-urban areas of Gelsha from which the study population was systematically selected. Mothers/caregivers and their under-five children who resided in semi-urban areas of Gelsha during the study period were included in the study. Under-five children who had been away from the study area during the 2 weeks prior to data collection but were available during data collection were excluded, because a child might develop acute diarrhea due to conditions in the other area where he/she had been before data collection.

### Sample size determination and sampling procedure

The sample size for this study was calculated using a single population proportion formula: [*Z*-_푎/2_]^2 *^P[1-*P*]/*d*^2^ [14] with the assumptions of 13.7% prevalence (*P*) of acute diarrhea among children under five in Amhara region, northern Ethiopia [8], 3% margin of error (*d*), *Z*_1_-_푎/2_ at 95% CI (confidence interval) = 1.96, and since source population of 770 under-five children in Gelsha was less than 10,000, sample size correction formula was used. We considered a 10% non-response rate and the final adequate sample size of the study was set at 340.

First, a preliminary assessment was conducted in order to identify households with at least one under-five child and the K interval was calculated. Then, the first household with an under-five child was selected by lottery method. Next, households with under-five children were selected using systematic sampling technique of every 2nd interval. In households with two or more children under five, one child was randomly selected and included into the study. Also, in a case where more than one caregiver of under-five children lived in the same household, only one caregiver was selected by a lottery method for the study. 

### Operational definitions

#### Acute diarrhea

Diarrhea among children under five was identified by asking the participants’ mothers/caregivers questions based on WHO-defined signs and symptoms of diarrhea [15] that had occurred during the previous 2 weeks. The WHO protocol [15] does not specify the recall period and the type of diarrhea. Because our study focused on acute diarrhea, we adopted a two-week recall period as specified in the World Gastroenterology Organization global guidelines for acute diarrhea surveys [16].

#### Good handwashing practice at critical times

Mothers/caregivers who reported washing their hands using water and soap at three or more of the five critical times: before preparing food, before feeding a child, before eating, after defecation and after cleaning a child who has defecated [17].

#### Poor handwashing practice at critical times

Mothers/caregivers who reported washing their hands using water and soap at two or fewer of the five critical times [17].

### Study variables

The dependent variable was acute diarrhea, denoted as yes [1] or no (0), where ‘yes’ indicated the presence of acute diarrhea and ‘no’ indicated the absence of acute diarrhea during the 2 weeks before to the survey.

The independent variables were socioeconomic/demographic, environmental and behavioral factors. Socio-demographic and economic variables that were self-reported by the study participants were mother’s/caregiver’s age (years), education status, marital status, household size (persons), house ownership, age of the child (months), child’s sex, birth order of child, total number of under-five children in the household and household economic status (wealth status). Wealth status of the study participant household was estimated using principal component analysis (PCA).

Environmental factors included water, latrine and waste-related characteristics. Water-related variables that were self-reported were: main source of drinking water, round-trip time taken to obtain water (minutes), **w**ater consumption per capita per day (liters). Latrine-related factors that were observed by the data collectors were: type of latrine facility, presence of feces around the pit hole/slab/floor of the latrine, presence of feces in the house compound and presence of flies on the floor and/or around the latrine facilities. Latrine ownership was measured by self-report of the study participants. Latrine distance from home (latrine proximity) was measured using GPS (geographical positioning system). Waste-related factors that were observed were presence of open raw sewage inside the house compound, presence of uncollected solid waste inside the house compound.

Behavioral factors that were self-reported by mothers/caregivers included child breastfeeding practices, child breastfeeding status (exclusive or partial), duration of breastfeeding, child given complementary food, age at which complementary food was started, rotavirus and measles vaccine status. Mothers’/caregivers’ hand- washing practice was computed as good or poor based on self-reported practice at the five critical times.

### Data collection and quality assurance

A structured questionnaire was prepared on acute diarrhea and associated factors in English and then translated to Amharic and then retranslated back to English, to ensure consistency of the questions. The questionnaire consisted of two parts. The first part included questions about the presence of acute diarrhea among the under-five children in the 2 weeks before the interview. The second part included questions about socio-demographic and economic, environmental, and behavioral factors.

Before the start of the actual data collection, the questionnaire was pretested on similar participants of 5% of the sample size in semi-urban areas of nearby Alansha. Some amendment was done based on the pre-test result before the actual data collection. The data were collected by face-to-face interviews and observations at the households from January to March, 2019. Four clinical nurses were recruited for data collection. They were given a day-long training session on study objectives, data collection tools, inclusion and exclusion criteria together with a work plan, and ethical issues.

Inter-observer reliability was ensured by providing clear definitions of variables to be recorded, by training data collectors, and by providing feedback about discrepancies during daily supervision. The principal investigator and trained supervisors were involved in the supervision of the overall data collection process. During the administration of the survey, each questionnaire was checked daily for completeness and consistency before the data entry. Unfilled or incompletely filled responses on a questionnaire were re-sought on the same or the next day by the same data collector.

In addition, to check the reliability of the information entered, 10% of the study participants were randomly selected and re-interviewed by another interviewer to check the reliability of the information entered by different interviewers. The qualifications of the interviewers and the training they received reduced the likelihood of interviewer bias. The data entries were checked for accuracy by reviewing randomly selected 10% of the questionnaires. In addition to this, descriptive statistics, such as cross-tabulations and frequency distributions were examined before performing statistical analysis.

### Data management and analysis

The collected data were checked, coded and entered into EpiData version 3.1 and exported to SPSS version 25.0 for data cleaning and analysis. Descriptive statistics (frequency distribution and cross-tabulations) were calculated for categorical variables and the mean with standard deviation was estimated for continuous variables. Data about households’ economic status was estimated by principal component analysis. The principal component analysis (PCA) was done to construct the household wealth index with the following considerations: communality value > 0.5, Kaiser-Meyer-Olkin (KMO) value > 0.5, and eigen-values greater than one [18]. Multicollinearity between independent variables was checked with the standard error of the coefficient with a cut-off point greater than 2 [19], which was not observed.

Bivariable (Crude Odds Ratio [COR]) and multivariable (Adjusted odds ratio [AOR]) analysis were employed using logistic regression analysis with a 95% confidence interval (CI). From the bivariable analysis, variables with *p < 0.25* were considered for multivariable analysis. From the multivariable logistic regression analysis, variables with a significance level at *p < 0.05* were taken as statistically significant and independently associated with acute diarrhea among under-five children. The model fitness was found to be good using the Hosmer Lemeshow goodness-of-fit test [19] and the model was fit at a *p*-value = 0.935.

## Result

### Socio-demographic characteristics

Out of 340 under-five children in the study, 335 participated, for a response rate of 98%. The majority, 201(60.0%) of the mothers/caregivers were literate. Homes were owned by more than three-fourths of the households 297 (88.7%) (Table 1).

**Table 1:** Mother/caregiver-related and other socio-economic and demographic factors in semi-urban areas of Gelsha, Northeast Ethiopia, January to March 2019

Variables	Number (*N* = 335)	Acute diarrhea		COR (95% CI)	*p*-value
	***n*** (***%***)	Yes	No		
		***n***	***n***		
**Age of mothers/caregivers (years)**					
< 25	45 (13.4)	6	39	1.05 (0.38–2.87)	0.927
25–34	165 (49.3)	15	150	0.37 (0.32–1.44)	0.313
> **35**	125 (37.3)	16	109	Ref	
**Mothers’**/**caregivers’ education status**					
**Illiterate**	134 (40)	22	112	2.44 (1.2–4.9)	0.012
**Literate**	201 (60)	15	186	Ref	
**Mothers’**/**caregivers’ marital status**					
Unmarried	61 (18.2)	7	49	1.18 (0.49–2.85)	
Married	274 (81.8)	30	249	Ref	
**Household size (persons)**					
≤ 5	235 (70.1)	24	211	Ref	
> 5	100 (29.2)	13	87	1.3 (0.64–2.7)	0.460
**House ownership**					
Own	297 (88.7)	32	265	Ref	0.240
Rent	38 (11.3)	5	33	1.25 (0.46–3.44)	0.180
**Wealth index**					
Poor	142 (42.4)	17	125	0.7 (0.28–1.73)	0.440
Medium	144 (43.0)	12	132	0.47 (0.18–1.22)	0.120
Rich	49 (14.6)	8	41	Ref	

*Ref* reference category, *COR* crude odds ratio, *CI* confidence interval

### Child-related socio-demographic factors

In this study, 128 (38.2%) children were in the age range of 36–59 months, and of the others, 36 (11.6%) were between 0 and 5 months, 42 (12.5%) between 6 and 11 months, 37 (11%) between 12 and 23 months, 89 (26.6%) between 24 and 35 months and 55.2% were female. More than three-fourths (83.90%) households had only one child under five (Table 2).

**Table 2:** Child-related socio-demographic factors in semi-urban areas of Gelsha, northeastern Ethiopia, January to March 2019

Variables	Number (*N* = 335)	Acute diarrhea		COR (95% CI)	*p*-value
		Yes	No		
	***n*** (***%***)	***n***	***n***		
**Age of the child (months)**					
0–5	36 (11.6)	5	34	2.54 (0.5–7-8.5)	0.130
6–11	42 (12.5)	9	33	4.71 (1.6–13.6)	0.004
12–23	37 (11.0)	11	26	7.3 (2.59–20.65)	0.001
24–35	89 (26.6)	5	84	1.09 (0.31–3.35)	0.962
36–59	128 (38.2)	7	121	Ref	
**Sex of the child**					
Male	150 (44.8)	14	136	Ref	
Female	185 (55.2)	23	162	1.37 (0.68–2.7)	0.37
**Birth order of the child**					
First	97 (29.0)	6	91	Ref	
Second	94 (28.1)	11	83	2.01 (0.71–5.67)	0.180
Third	59 (17.6)	8	51	2.38 (0.78–7.24)	0.130
Fourth	43 (12.8)	5	38	1.99 (0.57–6.94)	0.280
Fifth or above	42 (12.5)	7	35	3.03 (0.95–9.66)	0.600
**Total number of under-five children in the household**					
One	281 (83.9)	19	262	Ref	
Two or more	54 (16.1)	18	36	6.89 (3.31–14.34)	< 0.001

*Ref* reference category, *COR* crude odds ratio, *CI* confidence interval

### Environmental-related factors

Out of the total 335 households, two-thirds 213 (63.6%) of study participants used water from improved sources. Round-trip time taken to fetch water for two-thirds 224 (66.9%) was > 30 min (Table 3). On average, most 280 (83.6%) of the households used an improved latrine. Most of the latrines were private 281 (83.9%) and almost two-thirds 206 (61.5%) of the latrines were 15 m or more from home (Table 4).

**Table 3:** Water-related factors in semi-urban areas of Gelsha, northeastern Ethiopia, January to March 2019

Variables	Number (*N* = 335)	Acute diarrhea		COR (95% CI)	*p*-value
		Yes	No		
	***n*** (***%***)	***n***	***n***		
**Main sources of drinking water**					
Improved	213 (63.6)	11	202	Ref	
Unimproved	122 (36.4)	26	96	4.97 (2.36–10.48)	< 0.001
**Round-trip time to obtain water (minutes)**					
< 30	111 (33.1)	6	105	Ref	
> 30	224 (66.9)	31	193	2.81 (1.13–6.95)	0.025
**Water consumption per capita per day (liters)**					
< 20	254 (75.8)	29	225	1.17 (0.51–2.68)	0.700
> 20	81 (24.2)	8	73	Ref	

*Ref* reference category, *COR* crude odds ratio, *CI* confidence interval

**Table 4:** Latrine- and waste-related factors in semi-urban areas of Gelsha, northeastern Ethiopia, January to March 2019

Variables	Number (*N* = 335)	Acute diarrhea		COR (95% CI)	*p-*value
		Yes	No		
	***n (%)***	***n***	***n***		
**Type of latrine facility**					
Improved	280 (83.6)	31	249	Ref	
Unimproved	55 (16.4)	6	49	0.98 (0.39–2.48)	0.970
**Ownership of latrine**					
Private	281 (83.9)	31	250	Ref	
Shared	54 (16.1)	6	48	1.01 (0.39–2.55)	0.980
**The proximity of latrine facility to home (meters)**					
< 15	129 (38.5)	14	115	0.97 (0.48–1.96)	0.930
> 15	206 (59.7)	23	183	Ref	
**Presence of feces around the pit hole/slab/floor of the latrine**					
Yes	135 (40.3)	23	112	2.72 (1.35–5.52)	0.005
No	200 (59.7)	14	186	Ref	
**Presence of feces in the** **house** **compound**					
Yes	48 (14.3)	11	37	2.98 (1.36–6.54)	0.006
No	287 (85.7)	26	261	Ref	
**Presence of flies on the floor and/or around the latrine facilities**					
Yes	82 (24.5)	10	72	1.16 (0.54–2.52)	0.701
No	253 (75.5)	27	226	Ref	
**Presence of open raw sewage inside the house compound**					
Yes	55 (16.4)	8	47	1.47 (0.63–3.42)	0.360
No	280 (83.6)	29	251	Ref	
**Presence of uncollected solid waste inside the** **house** **compound**					
Yes	132 (39.4)	16	116	1.14 (0.59–2.38)	0.610
No	203 (60.6)	21	182	Ref	

*Ref* reference category, *COR* crude odds ratio, *CI* confidence interval

### Behavioral-related factors

Among 335 mothers/caregivers, the majority 184 (54.9%) practiced poor handwashing, while 151 (45.1%) practiced good handwashing. Of the total 335 children, about half 177 (52.8%) had never been breastfed and only 44 (13.1%) of the children were exclusively breastfed. One-fifth 65 (21.7%) of children started complementary food at less than 6 months of age. Less than one-tenth 24 (7.2%) of the children had not been given a rotavirus vaccine and one-fifth 70 (20.9%) of children also had not been given a measles vaccine (Table 5).

**Table 5:** Behavioral-related factors in semi-urban areas of Gelsha, northeastern Ethiopia, January to March 2019

Variables	Number (*N* = 335)	Acute diarrhea		COR (95% CI)	*p*-value
		Yes	No		
	***n*** (***%***)	***n***	***n***		
**Mothers’/caregivers’ hand washing practice at critical times**					
Poor	184 (54.9)	22	162	1.23 (0.61–2.46)	0.560
Good	151 (45.1)	15	136	Ref	
**Child breastfeeding**					
Yes	158 (47.2)	20	138	Ref	
No	177 (52.8)	17	160	0.73 (0.37–1.45)	0.370
**Child breastfeeding status**					
Exclusive	44 (13.1)	5	39	Ref	
Partial	115 (34.5)	15	100	1.17 (0.39–3.44)	0.770
**Duration of breastfeeding**					
< 2 years	200 (59.7)	25	175	0.68 (0.33–1.41)	0.300
> 2 years	135 (40.3)	12	123	Ref	
**Child given complementary food**					
Yes	296 (88.4)	23 (6.8)	72 (21.5)	1.09 (0.36–3.28)	0.870
No	39 (11.6)	14 (4.2)	226 (67.5)	Ref	
**Age at which complementary food was started**					
Less than 6 month	65 (21.7)	10 (3)	55 (16.4)	1.59 (0.72–3.52)	0.250
6 month or older	234 (78.3)	24 (7.2)	210 (62.6)	Ref	
**Rotavirus vaccine given**					
Yes	311 (92.8)	31 (9.2)	280 (83.6)	Ref	
No	24 (7.2)	6 (1.8)	18 (5.4)	3.01 (1.18–14)	0.030
**Measles vaccine given**					
Yes	265 (79.1)	25 (7.5)	240 (71.6)	Ref	
No	70 (20.9)	12 (3.6)	58 (17.3)	1.98 (0.94–4.18)	0.071

*Ref* reference category, *COR* crude odds ratio, *CI* confidence interval

### Prevalence and associated factors of acute diarrhea

The prevalence of acute diarrhea among the under-five children was 11.0% (95%CI: 7.8–14.3%). From the multivariable logistic regression analysis, child’s age between 12 and 23 months, presence of two or more children under five in the house, unimproved water sources and presence of feces around the pit hole/slab/floor of the latrine showed significant association with the occurrence of acute diarrhea among under-five children.

A child aged between 12 and 23 months was 4.85 times more likely to develop acute diarrhea than those in other-age categories (AOR = 4.68, 95%CI: 1.45–1.5). Age groups 0–5 months, 6–11 months, 24–35 months and 36–49 months did not show significant association with the occurrence of acute diarrhea. The presence of two or more children under-five in the house also showed a significant association with acute diarrhea. Children in households with two or more children under-five were 2.84 times more likely to develop acute diarrhea than those in households with only one child (AOR = 2.84, 95%CI: 1.19–6.81) (Table 6).

**Table 6:** Multivariable analysis of acute diarrhea among under-five children in semi-urban areas of Gelsha, northeastern Ethiopia, January to March 2019

Variables	Acute diarrhea		COR (95% CI)	AOR (95% CI)	*p-*value
	Yes	No			
	***n***	***n***			
**Age of the child (months)**					
0–5	22	162	2.54 (0.5–7-8.5)	1.12 (0.28–4.48)	0.675
6–11	15	136	4.71 (1.6–13.6)	1.69 (0.51–5.58)	0.431
12–23	5	39	7.3 (2.59–20.65)	4.68 (1.45–15.0)	< 0.001
24–35	20	138	1.09 (0.31–3.35)	0.74 (0.21–2.62)	0.984
36–59	17	160	Ref	Ref	
**Total number of under-five children in household**					
One	19	262	Ref	Ref	
Two or more	18	36	6.89 (3.31–14.34)	2.84 (1.19–6.81)	0.001
**Main sources of drinking water**					
Improved	11	202	Ref	Ref	
Unimproved	26	96	4.97 (2.36–10.48)	2.97 (1.28–6.87)	0.001
**Presence of feces around the pit hole/slab/floor of the latrine**					
Yes	23	112	2.72 (1.35–5.52)	3.34 (1.34–8.31)	< 0.001
No	14	186)	1	1	

*Ref* reference category, *COR* crude odds ratio, *AOR* adjusted odds ratio, *CI* confidence interval

On the other hand, children from households that used water from unimproved sources were almost three times more likely to develop acute diarrhea than those in households who used water from improved sources (AOR = 2.97, 95%CI: 1.28–6.87). Similarly, children in households with feces observed around the pit hole/slab/floor of the latrine were 3.34 times more likely to develop acute diarrhea than those in households with no feces seen around the pit hole/slab/floor of the latrine (AOR = 3.34, 95%CI: 1.34–8.31) (Table 6).

## Discussion

This community-based cross-sectional study was conducted with the aim to determine the prevalence of and factors associated with acute diarrhea among under-five children in semi-urban areas of Gelsha. The prevalence of acute diarrhea among under-five children was 11% and was significantly associated with a child’s age between 12 and 23 months, the presence of two or more under-five children in the house, unimproved water sources, and the presence of feces on the pit hole/slab/floor around the latrine facility.

This prevalence of 11% is comparable with the 12% reported in the Ethiopian DHS in 2016. In the context of Sustainable Development Goals, over the last 3 years, situations have not progressed much. This finding is also consistent with studies conducted in Dangla district (open-defecation-free *kebeles* 9.9%) [20], Wolita Soddo town (11%) [9], in Sidama Zone (13.6%) [21] and among non-model households in Hawassa (14.0%) [22]. This similarity of prevalence from different studies in Ethiopia might be due to the similarity of the water, sanitation and hygiene practices.

The prevalence is also much lower than found in studies conducted in several areas in Ethiopia such as in Debre Berhan Town 16.4% [23], in Sheka Zone 21.8% [24], in North Gondar Zone 22.1% [25], 17.2 and 23.2% in Goba District among ODF (open defecation-free) and non-ODF households respectively [26], in Harena Buluk Woreda 28.4% [27], in Wonago District 30.9% [28], in Uganda (29.1%) [29], in Yemen 29.07% [30], in Benishangul Gumuz Regional State 22.1% [31], in Arba-Minch District 30.5% [32], among the nomadic people in Hadaleala District 26.1% [33], in Tiko-Cameroon 23.8% [34], rural Burundi 32.6% [35], Senegal 26% 2014) [6], Enderta Wereda (35.6%) [13], Nekemte town 28.9% [36], Kersa District 22.5% [13], Mecha District 22.1% [37], Jabithennan District 21.5% [38] and in Ethiopia 15.6% [39]. The lower prevalence of acute diarrhea in our study might be due to the strong provision of urban water, sanitation and hygiene (WASH) conditions in Ethiopia, including in Gelsha peri-urban areas. Also, the difference may be a result of the proper implementation of a health extension program in Gelsha, which is important for disease prevention, compared to other areas.

This study showed that the odds of contracting acute diarrhea were higher in children aged between 12 and 23 months. At this age, children begin walking and are at high risk of contamination from contact with different environmental sources. This finding is consistent with the studies conducted in Sidama Zone [21], in Benishangul Gumuz Regional State [31], in Bangladesh [35], in Wolita Soddo town, in Mecha District [37], in Hadaleala District [33], in Arba-Minch District [32], a study conducted by Beyene et al. among agricultural and agro-pastoralist communities of Southern Ethiopia [40] and the Ethiopian DHS 2016 report [8]. However, this finding is in contrast with the studies conducted in Tigray [41], Wolita Soddo town [9] and Farta Wereda [42]. This difference might be due to the children’s mothers/caregivers different educational levels in these areas.

This study also showed that acute diarrhea among under-five children had a significant association with the presence of two or more under-five children in the house. Children in households with two or more children in this age group were 2.84 times more likely to develop acute diarrhea than those in households with only one such child. This might be due to reduced care given to the child by a mother with more under-five children. This finding is in agreement with studies conducted in Northeast Ethiopia, Tigray [41] Benishangul [31], Shebedino District [43], Yaya Gulele District [44], Wolita Soddo Town [9], in Hadaleala District [33]. However, a study that was conducted in Arba-Minch District [32] did not find any significant association between acute diarrhea and the number of children. Differences might be due to increased care given to the child by the mothers/caregivers in these areas.

In this study, children from households that  got water from unimproved sources were almost three times more likely to develop acute diarrhea than those in households that  got water from improved sources. Exposure to different pathogens from contaminated water might cause this acute diarrhea. This finding was supported by the studies conducted in Uganda [29], in Sheka Zone [24], Indonesia [45], Nigeria [46], Amhara Region [47], Tigray [9], Derashe District [48], Jigjiga District [49] but is in contrast to studies conducted in Farta Wereda [42], Benishangul Gumuz [31] and Debre Berhan town [50]. This difference might be due to the use of water from mixed improved and unimproved sources and the use of different water treatment practices.

Acute diarrhea among under-five children showed significant association with the presence of feces around the pit hole/slab/around the latrine facility. Children from households with feces seen around the pit hole/slab/around the latrine were three times more likely to develop acute diarrhea than children from households with a clean latrine. This may be due to the presence of feces around the pit hole/slab/around the latrine creating conditions for the breeding of flies that allows the transmission of diarrhea-causing pathogens. This finding was consistent with former studies in rural area of North Gondar [51], Debre Berhan town [50], Nekemte town [36], and Gummer Wereda [52] but is in contrast with studies conducted in Bahirdar [53], and Derashe District [48]. This difference might be due to a difference in keeping the cleanliness of the sanitation facility.

### Limitations of the study and gaps for future research

There are several limitations to this study. As data were collected from self-reported questionnaire or direct observation, there was risk of response bias with over/under reporting of acute diarrhea. This has also happened in other studies from Ethiopia [54]. Our findings do not reveal seasonal variation in prevalence since the study was conducted from January to March, which is a dry season. Other studies have shown that during the rainy season diarrhea is increased [55]. Besides, the prevalence was determined based on the reports of mothers/caregivers without confirmation by laboratory examination. Thus, further study including laboratory examination of stool and water samples is needed to measure factors related to acute diarrhea more accurately.

Because there are several residual confounding factors due to unmeasured variables, additional studies are highly recommended that consider water, sanitation, and hygiene management status, which will enable collection of strong evidence. The other limitation of the study was that the study did not investigate household, individual, and community factors using multilevel analysis and the fact that there was a scarcity of literature on semi-urban areas; thus, the discussion compared findings with urban and rural community studies. Despite these limitations, the study provides new insight into the extent of acute diarrhea among under-five children in semi-urban areas.

## Conclusion

This study identified socio-economic, environmental, and behavioral factors associated with acute diarrhea among under-five children in semi-urban areas of Gelsha. The study revealed that the prevalence of acute diarrhea among these children was 11%, a result that indicates a relatively high prevalence in these areas. The main factors that were significantly associated with acute diarrhea were a child’s age between 12 and 23 months, the presence of two or more under-five children in the house, unimproved water sources, and the presence of feces around the pit hole/slab/floor of the latrine**.** Implemention of various prevention strategies is essential, such as the provision of health education to mothers/caregivers that focuses on keeping sanitation facilities clean and child care, and construction of improved water sources. Furthermore, implementing a strong health extension program (HEP), advocating open defecation-free (ODF) environment, and practicing community-led total sanitation and hygiene (CLTSH) approach might be helpful to sustainably reduce childhood diarrhea. Hence, collaborative work is urgently needed among the communities, government organizations, and civic associations to prevent acute diarrhea among under-five children in semi-urban areas of Ethiopia.

## Supplementary Information

**Additional file 1.**

## Data Availability

The datasets collected and/or analyzed during the current study are available from the corresponding author on reasonable request.

## Abbreviations

AOR: Adjusted Odds Ratio
CI: Confidence Interval
COR: Crude Odds Ratio
DHS: Demographic and Health Survey
NGOs: Non-Governmental Organizations
SPSS: Statistical Package for Social Sciences
