# Supplementary material for: Prevalence of acute diarrhea and associated factors among children under five in semi-urban areas of northeastern Ethiopia
Source: BMC Pediatr. 2021 Jun 26;21:290. doi: 10.1186/s12887-021-02762-5 (PMC8235618; doi:10.1186/s12887-021-02762-5)
Supplement: Supplementary file 1 — Additional file 1. [file 12887_2021_2762_MOESM1_ESM.docx]

English version of the questionnaire

**Questions to household survey of the study**

Questionnaire ID_________________

Name of semi urban district_____________

Date of data collection________________

Data collector name ____________________signature_________________

Supervisor name ______________________ signature_________________

Completed/uncompleted questionnaires Completed ______ Uncompleted _______

**Instruction: Circle the respondents answer from the given alternatives and write respondents answer on the blank space.**

**Part 1(100): - Socio-demographic factors**

| **Code** | Questions | Response options | **Skip** |
| --- | --- | --- | --- |
| 101 | Age of mother/caregiver ( in years) | _________ |  |
| 102 | Religion of mother/caregiver | __________ |  |
| 103 | Ethnicity of mother /caregiver | __________ |  |
| 104 | Sex of the respondent | __________ |  |
| 105 | Educational status of mother/caregiver | __________ |  |
| 106 | Marital status of the mother/caregiver | 1.Single  2.Married  3.Widowed  4.Divorced |  |
| 107 | Household size | __________persons |  |
| 108 | House ownership | 1.Private/owned  2.Rent from Kebelle  3.Rent from private  4.Niether rented nor owned |  |
| 109 | Age of the child (in months) | __________ |  |
| 110 | Sex of the child | 1.Male  2.Female |  |
| 111 | Birth order of the child | __________ |  |
| 112 | Total number of under five children | __________ |  |
| 113 | Is the child showed the following symptoms in the past two weeks? (more than one answer is possible ) | 1.Had at least three and/or more times watery loose stool per day, and/or stool with mucus  2. Fever  3.Thirsty  4. Vomiting  5.Refuses to eat/drink/ take breast  6.Bloody stools  99.Other (specify)________ |  |
| 114 | Do the child had acute diarrhea during the past two weeks? (Filled by data collector based on Q 116) | 0.No  1.Yes |  |
|  | **Wealth index questions** |  |  |
|  | **Urban wealth index** |  |  |
| 115 | Number of rooms | __________ |  |
| 116 | Do you have separated bedroom? | 0.No  1.Yes |  |
| 117 | Do you have separated kitchen? | 0.No  1.Yes |  |
| 118 | From which material your house floor is made? (more than one answer is possible ) | 1.Natural ground  2.Muck/smooth by cows faces  3.Wood  4.Cement  5.If others list __________ |  |
| 119 | From which material your house roof is made? | 1.Grass/ leaf  2.Corrugated iron  99.Other/specify__________ |  |
| 120 | From which material your house wall is made? (more than one answer is possible ) | 1.Wood but not have mod  2.Wood with mod  3.Wood and cement  4.Blocket  99.If others list__________ |  |
| 121 | What is your energy source for food cooking? (more than one answer is possible ) | 1.Electricity system  2.Gas /kerosene  3.Wood /leaf  4.Charcoal  5.Animal faeces  99.If other list __________ |  |
| 122 | Among the following materials, which one do you own? (more than one answer is possible ) | 1.Radio  2.Television  3.House phone  4.Fridge  5.Chair  6.Table  7.Bed and mattress which made from cotton spring  8.Mobile  9.Cycle  10.Motor cycle  11.Horse’s cart  12.Bajaj/car  13.Bank book  99.If other list __________ |  |
|  | **Rural wealth index** |  |  |
| 123 | Among the following materials, which one do you own? (more than one answer is possible ) | 1.Watch    2.Sofa  3.Chair  4.Table  5.Bed and mattress which made from cotton spring  6.Horse’s Cart  99.If others (specify) __________ |  |
| 124 | Do you have your own farm for the purpose of agriculture/cropping? | 0.No  1.Yes |  |
| 125 | From the following household animal do you have? (more than one answer is possible ) | 1.Ox/cow  2.Horse/donkey/ mule  3.Goat  4.Sheep  5.Hen  6.Beehive  99.Others (specify) __________ |  |

**Part II (200):-Environmental related variables**

| Code | | **Questions** | **Response** | | | **Skip** | | | |
| --- | --- | --- | --- | --- | --- | --- | --- | --- | --- |
| **Water related questions** | | | | | | | | | |
| 201 | | What is the main source of drinking water for members of your household? | 1.Tap water  2.Public tap  3.Protected well  4.Unprotected well  5.Protected spring  6.Unprotected spring  99.Other(specify) __________ | | | |  | | |
| 202 | | How long it take to reach the water source (Two-way trip)? | __________ minutes | | | |  | | |
| 203 | | Amount of daily water consumption for drinking, cooking and personal hygiene | __________liter | | | |  | | |
| **Latrine related questions** | | | | | | | | | |
| 204 | | Type of latrine facility  (If necessary ask permission to observe the facility) | | 1.Simple pit latrine  2.Ventilated improved pit latrine(VIP)  3.Pour-flush latrine  99.Other/specify ______ | | | |  | |
| 205 | | Ownership of latrine? | | 1.Privately owned  2.Shared  99.Other/specify _______ | | | |  | |
| 206 | | Proximity of latrine facility from home | | __________meters | | | |  | |
| 207 | | Feces seen around the pit hole/slab/floor of latrine (observation) | | 0.No  1.Yes | | | |  | |
| 208 | | Feces seen in the house compound  (observation) | | 0.No  1.Yes | | | |  | |
| 209 | | Flies observed on the floor and/or around the latrine facilities  (observation) | | 0.No  1.Yes | | | |  | |
| **Waste disposal related questions** | | | | | | | | | |
| 210 | Is there open raw sewage seen inside the compound during data collection? (Observation) | | | | 0.No  1.Yes | | | |  |
| 211 | Is there uncollected garbage (solid waste/refuse) seen inside the compound during data collections? (Observation) | | | | 0.No  1.Yes | | | |  |

**Part III (300):- Behavioral related variables**

| **Code** | Questions | Response options | **Skip** |
| --- | --- | --- | --- |
|  | Hand washing at five recommended times |  |  |
| 300 | Mother/caregiver hand washing with soap in the previous two weeks (more than one option is possible)  1.Before child feeding  2.Before food preparing  3.After cleaning a defecated child  4.After latrine use  5.Before eating | 0.No 1.Yes  0.No 1.Yes  0.No 1.Yes  0.No 1.Yes  0.No 1.Yes |  |
| 301 | Do you feed breast to your child? | 0.No  1.Yes |  |
| 302 | If yes for 301 what is your current breast feeding status? | 1. Exclusive  2. Partial |  |
| 303 | For how long have you been feeding breast to your child? | _________ |  |
| 304 | Does your child start complementary food? | 0.No  1.Yes |  |
| 305 | When do you introduce complementary food to your child? | _________ |  |
| 306 | Do your Child received Rota  Vaccination? | 0.No  1.Yes |  |
| 307 | Do your Child received measles  Vaccination? | 0.No  1.Yes |  |

THANK YOU FOR YOUR PARTICIPATION!!!
